# Supplementary material for: Protocadherin 15 suppresses oligodendrocyte progenitor cell proliferation and promotes motility through distinct signalling pathways
Source: Commun Biol. 2022 May 30;5:511. doi: 10.1038/s42003-022-03470-1 (PMC9151716; doi:10.1038/s42003-022-03470-1)
Supplement: Supplementary file 1 — Supplementary Data 1 [file 42003_2022_3470_MOESM1_ESM.pdf]

Supplementary Data 1
Source data underlying graphs presented in main figures of Zhen et al.

Figure 2c

Control shRNA	Pcdh15 shRNA
100	3.611237
100	6.787354
100	0.698297
100	4.823397

Figure 2g

control shRNA	Pcdh15 shRNA	No transfection
12.28739	7.522362	12.383
11.66062	7.463904	12.02018
10.76658	7.097379	10.53164

Figure 2l

Control shRNA	Pcdh15 shRNA-A	Pcdh15 shRNA-B	Pcdh15 shRNA-C
14.29326	10.61481	10.04163	11.5713
14.84547	9.238454	10.27592	10.46345
14.54202	9.44653	10.22494	9.306022

Figure 3g

Control-shRNA	Pcdh15-shRNA
10.3515	22.6831
11.1305	22.9133
9.0827	12.9862

Figure 3h

Control shRNA	pcdh15 shRNA-A	pcdh15 shRNA-B	pcdh15 shRNA-C
28.4295	46.86005	46.5593	46.5093
16.3274	34.29	35.2913	34.1455
26.5363	42.5659	40.8625	47.5235

Figure 3q

	control-shRNA	control-shRNA	control-shRNA	pcdh15-shRNA	pcdh15-shRNA	pcdh15-shRNA
cell in single	87.63152	91.44444	88.60778	81.8444	80.2758	82.86748
cell in pairs	10.5503	8.555556	10.11728	16.3699	19.7242	15.91234
cell in	1.818182	0	1.274945	1.78571	0	1.46969
cell in fours	0	0	0	0	0	0.126667

Figure 3r

control-shRNA	Pcdh15-shRNA
59.24787	36.83169
46.93185	32.64447
57.21912	38.94054

Figure 4b

control-shRNA	pcdh15 shRNA

1.09187	0.889082
0.645817	0.366404
1.341283	0.55349

Figure 4c

control shRNA	pcdh15 shRNA
0.771146	0.856694
0.67369	0.763281
0.540401	0.544781

Figure 4e

p-ERK1/ERK1		p-ERK2/ERK2	
control-shRNA	pcdh15-shRNA	control-shRNA	pcdh15-shRNA
0.398606	0.826693	1.138381	1.504537
0.305064	0.747518	1.340477	1.943964
0.314589	0.610468	1.246828	1.563034

Figure 4g

p-ERK1/ERK1				p-ERK2/ERK2			
Control-shRNA	PCDH15-shRNA	Control-shRNA+U0126	PCDH15-shRNA+U0126	Control-shRNA	PCDH15-shRNA	Control-shRNA+U0126	PCDH15-shRNA+U0126
100	199.1318	27.326	33.5428	100	113.898	14.6426	17.4034
100	139.4453	21.4031	34.5838	100	136.273	19.7364	26.837
100	151.1525	41.7225	26.2476	100	123.351	50.3355	32.4373

Figure 4l

Control-shRNA	PCDH15-shRNA	Control-shRNA+U0126	PCDH15-shRNA+U0126
16.931	25.139	4.05	6.3579
17.72512	28.7604	5.895	7.4252
17.5068	37.8791	8.5	10.87

Figure 5b

Control-shRNA	PCDH15-shRNA
1.873	18.38
4	56.6
4.974	41.65

Figure 5d

Control-shRNA	PCDH15-shRNA
8.761	47.49
9.749	74.79
9.103	80.92

Figure 5l

control-shRNA	Pcdh15-shRNA
5.08	2.97561
5.647059	3.09375
5.047619	3.125

Figure 5h

control-shRNA	pcdh15-shRNA
1.545	0.905536
1.063	0.5588
1.069	0.52

Figure 5j

Control-shRNA	PCDH15-shRNA
0.729404	0.521671
0.615759	0.244931
0.696832	0.328145
0.839343	0.198792
0.394916	0.273334
0.418429	0.251302
0.575311	0.327045
0.46474	0.311375
0.691089	0.323119
0.533602	0.267841
0.511624	0.427618
0.384027	0.221029
0.910134	0.699511
0.548212	0.245337
0.594506	0.227001
0.490641	0.421604
0.429384	0.414105
0.333408	0.319863
0.429251	0.284084
0.40295	0.2331
0.282465	0.183919
0.40295	0.369606
0.445132	0.403833
0.558018	0.56803
0.493827	0.403833
0.697125	0.230524
0.516094	0.434959
0.710979	0.233674
0.630021	0.449943
0.521941	0.400747
0.728526	0.393749
0.362869	0.344492
0.33306	0.294658
0.475645	0.211936
0.619217	0.243251
0.471249	0.376114
0.384068	0.280528
0.630961	0.300622
0.227894	0.288544
0.434153	0.27862

0.611506	0.391122
0.465037	0.388096
0.469926	0.225201
0.293808	0.391122
0.57317	0.388096
0.604427	0.225201
0.423068	0.233533
0.429702	0.524742
0.601278	0.297774
0.564056	0.391483
0.47506	0.299881
0.470543	0.198792

Figure 5k

Control-shRNA	PCDH15-shRNA
43.0348	30.7786
36.3298	14.4509
41.1131	19.3605
49.5212	11.7287
23.3	16.1267
24.6873	14.8268
33.9434	19.2957
27.4197	18.3712
40.7742	19.0641
31.4825	15.8026
30.1858	25.2295
22.6576	13.0407
53.6979	41.2711
32.3445	14.4749
35.0758	13.3931
28.9478	24.8746
25.3337	24.4322
19.6711	18.8719
25.3258	16.7609
23.774	13.7529
16.6654	10.8512
23.774	21.8067
26.2628	23.8261
32.923	33.5138
29.1358	23.8261
41.1303	13.6009
30.4495	25.6626
41.9478	13.7868
37.1712	26.5466
30.7945	23.6441
42.983	23.2312
21.4093	20.325
19.6505	17.3848
28.063	12.5042
36.5338	14.3518

27.8037	22.1907
22.66	16.5512
37.2267	17.7367
13.4458	17.0241
25.615	16.4386
36.0789	23.0762
27.4372	22.8977
27.7256	13.2869
17.3347	23.0762
33.817	22.8977
35.6612	13.2869
24.961	13.7784
25.3524	30.9598
35.4754	17.5686
33.2793	23.0975
28.0286	17.693
27.7621	11.7287

Figure 5l	Control-shRNA	PCDH15-shRNA
	12.0326	11.9331
	15.8704	4.32618
	10.0038	5.22971
	14.8591	10.551
	2.68729	6.67173
	7.60079	4.32822
	9.15314	7.97266
	11.7053	1.22507
	13.4233	7.07753
	9.69279	1.13531
	8.42596	10.1632
	10.9614	4.87862
	6.59721	2.16309
	1.87917	7.10368
	9.57088	2.13019
	10.9227	7.04252
	17.3603	9.57181
	3.21388	2.22744
	4.22292	2.49655
	16.087	7.87125
	4.58187	8.45106
	16.087	11.2852
	6.26783	3.72295
	3.80504	6.02656
	2.71018	3.72295
	17.2357	7.93047
	2.92331	6.33508
	9.18395	1.73251
	4.79282	13.0146
	7.27437	15.8431

17.5404	4.05658
7.86789	9.09023
9.098	7.15568
17.6922	2.61063
6.68759	8.79807
12.0179	9.07079
14.0938	7.74803
6.51507	3.76304
6.63458	7.85778
11.616	5.89773
12.0502	7.79914
8.32158	8.37235
10.8383	6.85963
9.06787	7.79914
11.2696	8.37235
16.8461	6.85963
10.1727	9.02982
8.8111	10.8155
14.7309	6.29594
8.21803	11.942
18.3222	6.65981
9.83654	10.551

Figure 6b	Control-shRNA	PCDH15-shRNA	Control-shRNA+U0126	PCDH15-shRNA+U0126
	5.705714	22.86	5.174038	24.16806
	8.722685	29.85333	9.450792	26.9963
	3.873497	17.85	4.046912	19.58333

Figure 6c	Control-shRNA	PCDH15-shRNA	Control-shRNA+U0126	PCDH15-shRNA+U0126
	9.290361	47.84	11.69593	42.66667
	7.913836	44.19133	10.60482	31.30667
	10.35765	28.35556	9.992691	28.10767

Figure 6e	Control-shRNA	Pcdh15-shRNA	Contol-shRNA+U0126	Pcdh15-shRNA+U0126
	4.823529	2.962963	4.636364	2.857143
	4.642857	2.733333	4.714286	3.029412
	5	3.352941	4.809524	3.179487

Figure 6f	Control-shRNA	PCDH15-shRNA	Control-shRNA+U0126	pcdh15-shRNA+U0126
	1.9466666	0.911	2.041667	0.99
	1.97925	0.96666	2.041667	1.076388
	2.033333	0.776667	2	0.722222

Figure 7b

Control-shRNA	PCDH15-shRNA
100	132.8143
100	120.0394
100	137.8859

Figure 7c

Control-shRNA	Pcdh15-shRNA
0.38	0.355
0.366364	0.431818
0.374783	0.35381

Figure 7e

control-shRNA	pcdh15-shRNA
1.365062	1.324165
1.209761	1.69704
1.27969	1.931176

Figure 7f

control-shRNA	pcdh15-shRNA
0.540122	0.643673
0.610642	0.714134
0.657479	0.641702

Figure 7i

Control-shRNA	Pcdh15-shRNA
0.836139	1.056569
0.628896	0.978384
0.472723	0.920638

Figure 7j

Control-shRNA	Pcdh15-shRNA
0.202631	0.380655
0.235737	0.411465
0.289141	0.410022

Figure 7k

Control-shRNA	Pcdh15-shRNA
0.840919	1.135117
0.858036	1.155147
0.890574	1.115998

Figure 7l

Control-shRNA	Pcdh15-shRNA
0.668851	0.832201
0.651889	0.740218
0.536267	0.782073

Figure 7m

Control-shRNA	Pcdh15-shRNA
0.458196	0.867206
0.708471	0.960744
0.567472	0.862117

Figure 9n

control-shRNA			pcdh15-shRNA		
DMSO	CK666	ML141	DMSO	CK666	ML141
26.57596	20.96576	20.11114	36.12855	19.9112	19.548
25.90653	21.17888	22.12941	32.22067	20.8177	18.1251
26.64439	18.69732	18.55645	31.14857	18.6707	18.8994

Figure 9p

control-shRNA	Pcdh15-shRNA	Contol-shRNA+ML141	Pcdh15-shRNA+ML141
10.10686	29.91621	3.812338	7.737764
16.58435	34.03174	6.708333	7.988872
11.94896	30.65875	7.754167	8.571429

Figure 9q

control-shRNA	Pcdh15-shRNA	Contol-shRNA+ML141	Pcdh15-shRNA+ML141
16.94472	37.91539	7.382759	10.51533
14.92353	32.50688	8.451241	12.09779
17.452	44.95052	7.65055	13.53304

Figure 10b

Control-shRNA	Pcdh15-shRNA	Contol-shRNA+ML141	Pcdh15-shRNA+ML141
5.128205	3.135135	2.88	2.666667
4.608696	2.976744	2.952381	2.946429
4.896552	2.941176	3	2.909091

Figure 10c

control-shRNA	Pcdh15-shRNA	Contol-shRNA+ML141	Pcdh15-shRNA+ML141
2.975	1.813888	2.06714	2.1
2.85	1.616662	1.805556	1.805556
2.725	1.5	1.68333	2
