# Supplementary material for: Protocadherin 15 suppresses oligodendrocyte progenitor cell proliferation and promotes motility through distinct signalling pathways
Source: Commun Biol. 2022 May 30;5:511. doi: 10.1038/s42003-022-03470-1 (PMC9151716; doi:10.1038/s42003-022-03470-1)
Supplement: Supplementary file 2 — Supplementary Information [file 42003_2022_3470_MOESM2_ESM.pdf]

SUPPLEMENTARY FIGURES:

Title: Protocadherin 15 suppresses oligodendrocyte progenitor cell proliferation and promotes motility through distinct signalling pathways

Authors: Yilan Zhen[1], Carlie L Cullen[1], Raphael Ricci[1], Benjamin S Summers[1], Sakina Rehman[2], Zubair Ahmed[2], Antoinette Y. Foster[3], Ben Emery[3], Robert Gasperini[1, 4], Kaylene M Young[1#]

Affiliations:

[1] Menzies Institute for Medical Research, University of Tasmania, Liverpool St, Hobart, Australia, 7000

[2] Department of Otorhinolaryngology Head and Neck Surgery, School of Medicine, University of Maryland, Baltimore, MD, 21201, USA.

[3] Jungers Center for Neurosciences Research, Department of Neurology, Oregon Health & Science University, Portland, OR, 97239, USA.

[4] School of Medicine, University of Tasmania, Liverpool St, Hobart, Australia, 7000

[#] **Correspondence** and requests for information should be addressed to kaylene.young@utas.edu.au

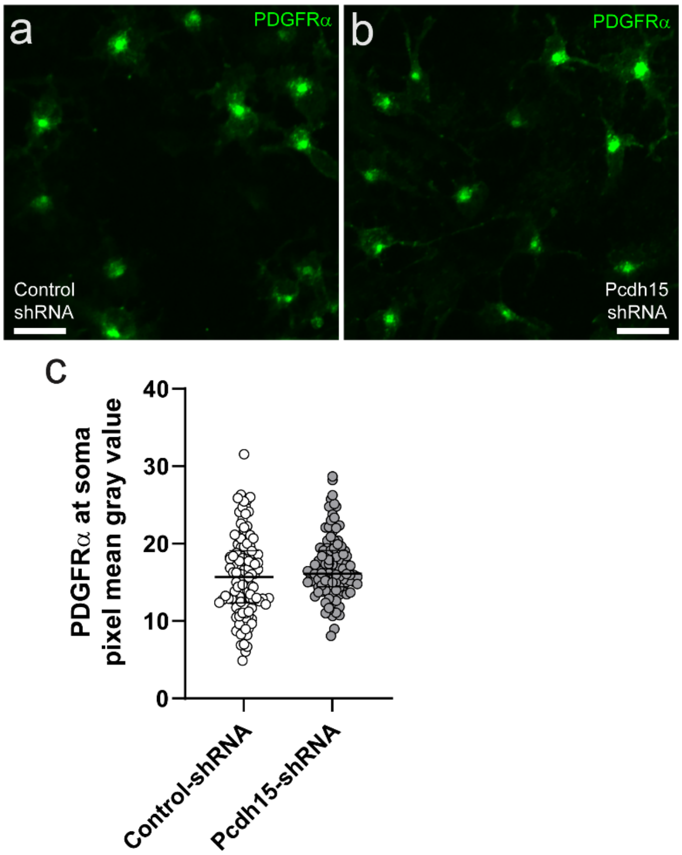

Supplementary Figure 1: PDGFRα expression is not grossly altered in OPCs treated with Pcdh15 shRNA

(a-b) Confocal images of control and Pcdh15 knockdown OPCs immunolabelled to detect PDGFRα (green). (c) Quantification of the mean gray value (sum of gray values for all pixels in the selection / total number of pixels) corresponding to PDGFRα labelling at the soma of OPCs in control and Pcdh15 knockdown cultures [median ± interquartile range, n ≥ 30 cells per treatment per culture across n=3 cultures, Mann whitney U test, U statistic = 4873, p = 0.08]. Scale bars represent 15µm.

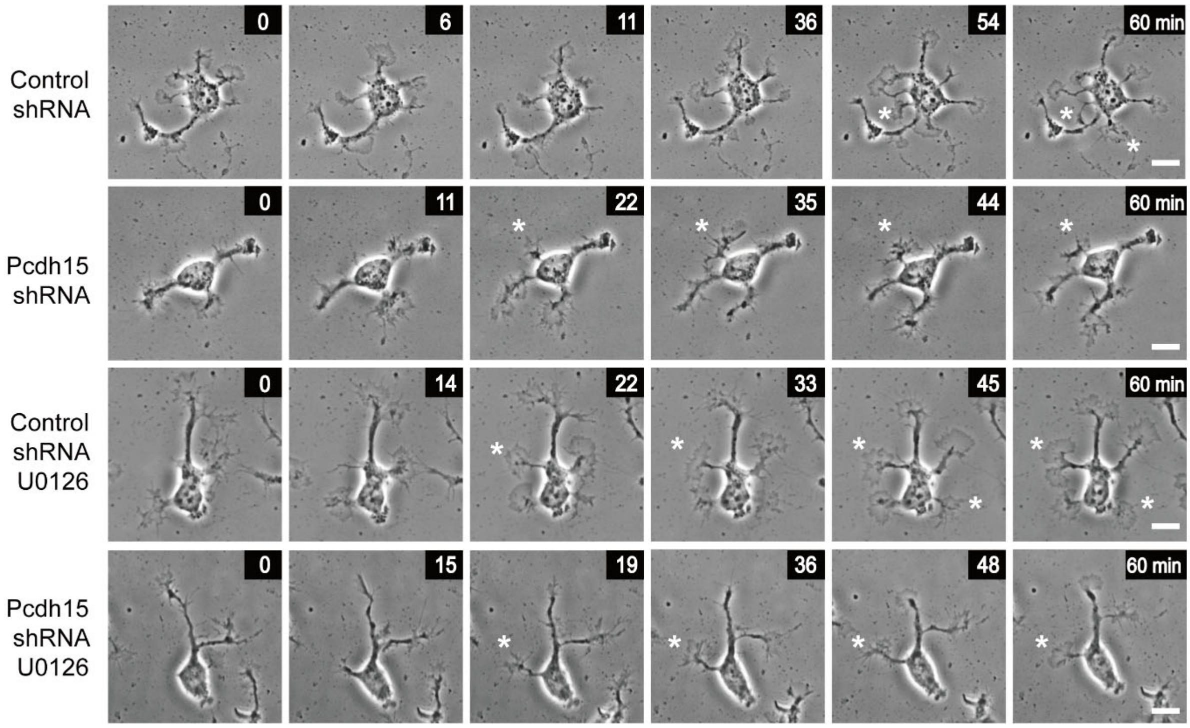

Supplementary Figure 2: Pcdh15 does not support process elaboration by suppressing ERK1/2 phosphorylation

Image series showing control and Pcdh15 knockdown OPCs, treated with DMSO or U0126, as they elaborate new processes. Images were collected every minute for 2 h to generate time-lapse videos for analysis. Individual image series are excerpts from the time-lapse videos, selected as discrete examples of process generation. All images are time-stamped relative to the first image depicted in the series (designated as 0 min). White asterisk denotes newly elaborated process. Scale bars represent 10µm.

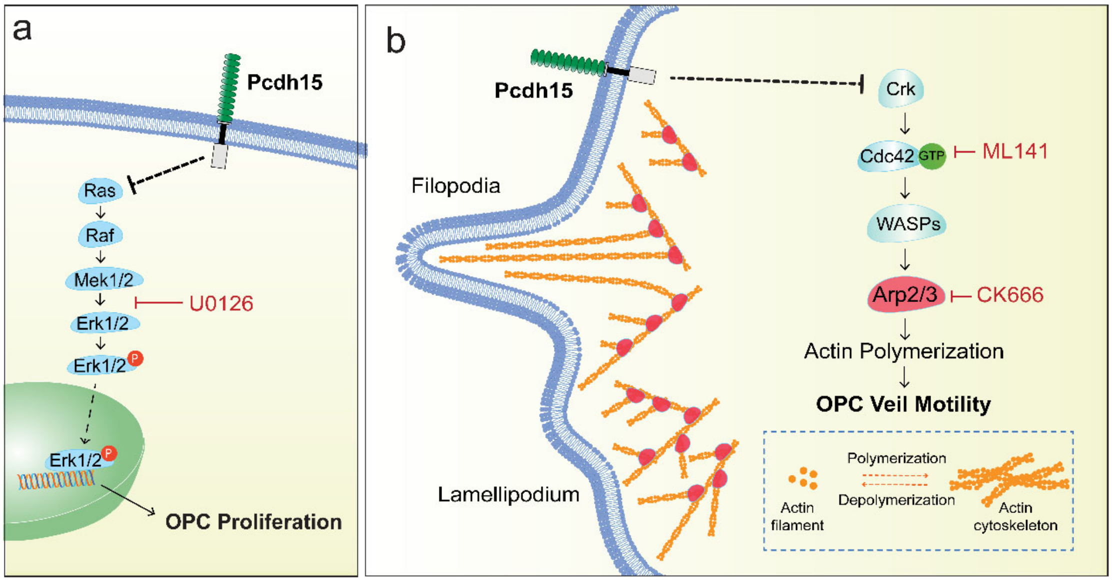

Supplementary Figure 3: Pcdh15 simultaneously suppresses ERK phosphorylation to reduce OPC proliferation and Cdc42 activity to promote rapid filopodial repulsion and dynamic veiling.

(a) Pcdh15 suppresses ERK phosphorylation to reduce OPC proliferation. (1) When Pcdh15 expression is knocked down in OPCs, p-ERK1/2 activity is increased. (2) When ERK1/2 phosphorylation is prevented by MEK1/2 inhibition with U0126, Pcdh15 knockdown cannot increase OPC proliferation. (b) Pcdh15 enhances the motility of actin-rich filopodia and veils. (1) When Pcdh15 expression is knocked down in OPCs, signalling via the cdc42-Arp2/3 signalling pathway is increased and filamentous actin accumulates within OPC veils. (2) Pharmacologically blocking Cdc42 GTPase activity (ML141) or Arp2/3 activity (CK666), reduces the amount of filamentous actin in OPC veils. (3) Pharmacologically blocking Cdc42 GTPase activity (ML141) restores veiling kinetics and filopodial self-repulsion to Pcdh15 knockdown OPCs. ----I indicates that Pcdh15 is a negative regulator of the MAPK signalling and Cdc42-Arp2/3 signalling pathways.

Figure 2a

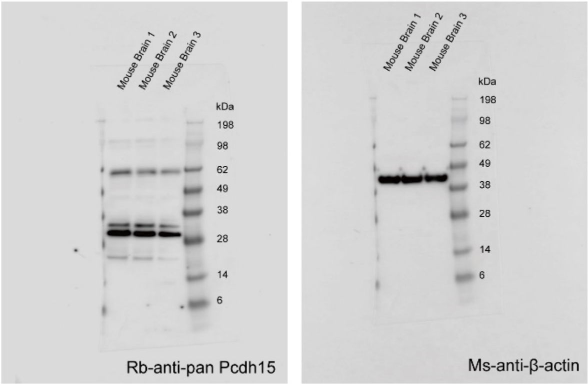

Figure 2b

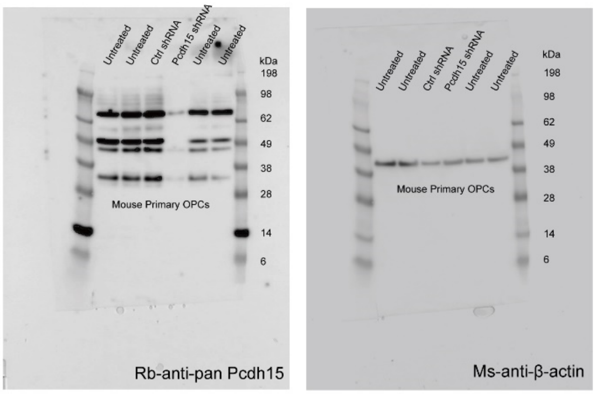

Figure 4a

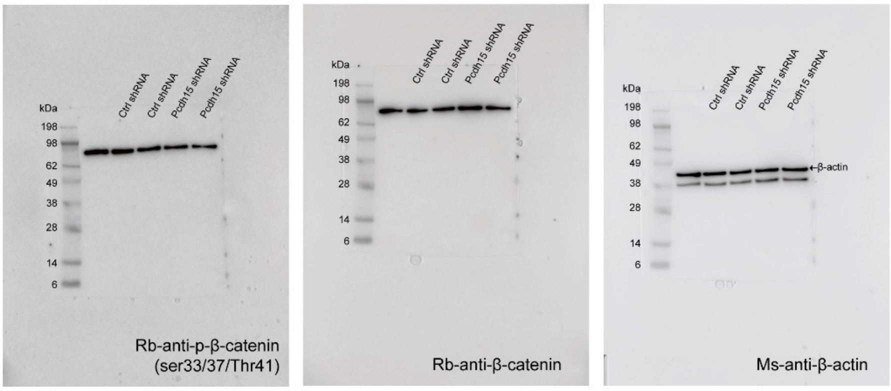

Figure 4d

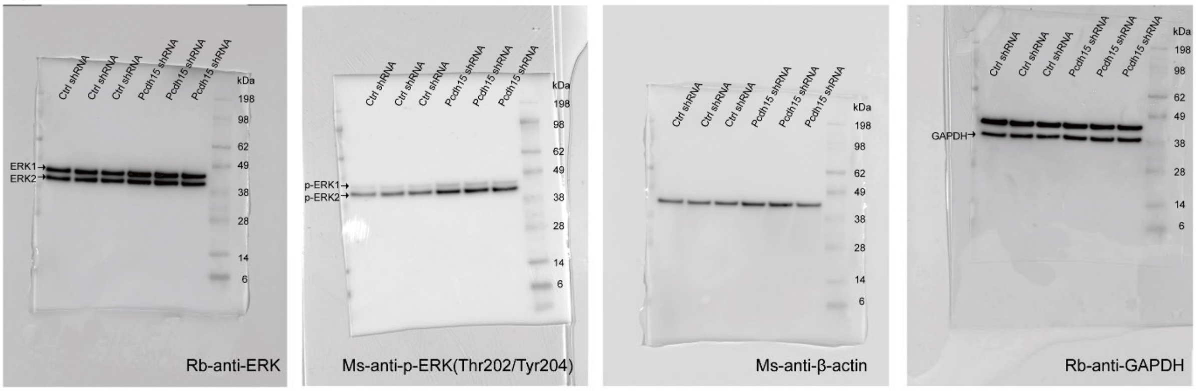

Figure 4f

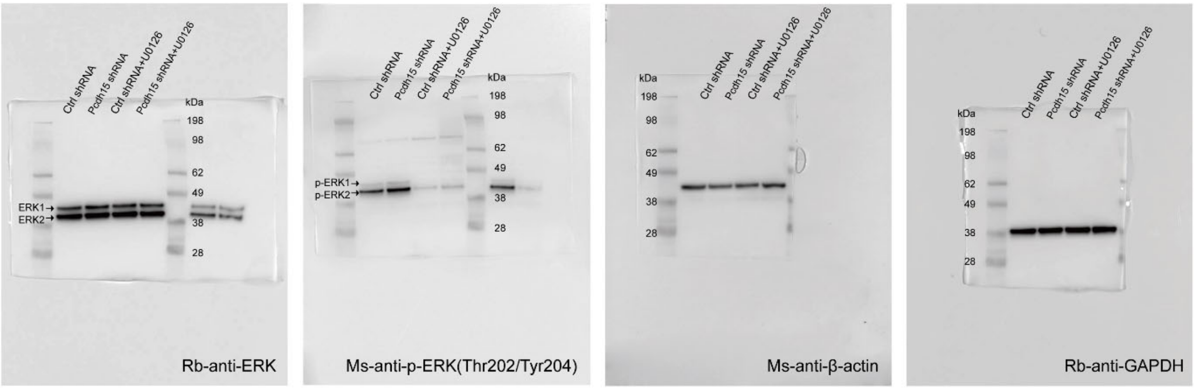

Supplementary Figure 4: Uncropped images corresponding to western blots in the main figures.

Figure 7d

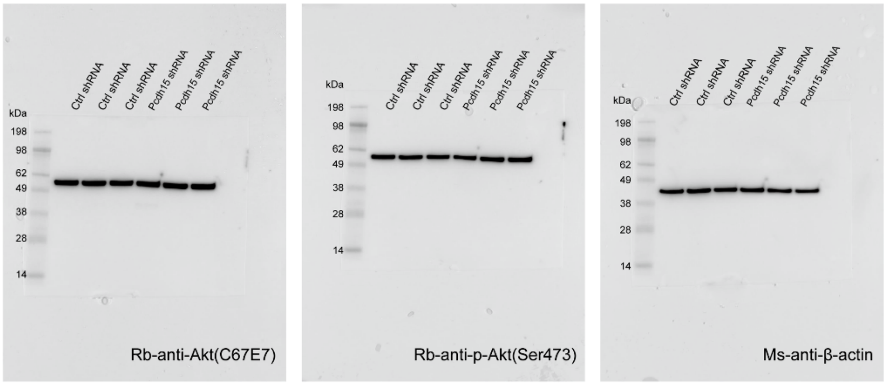

Figure 7h

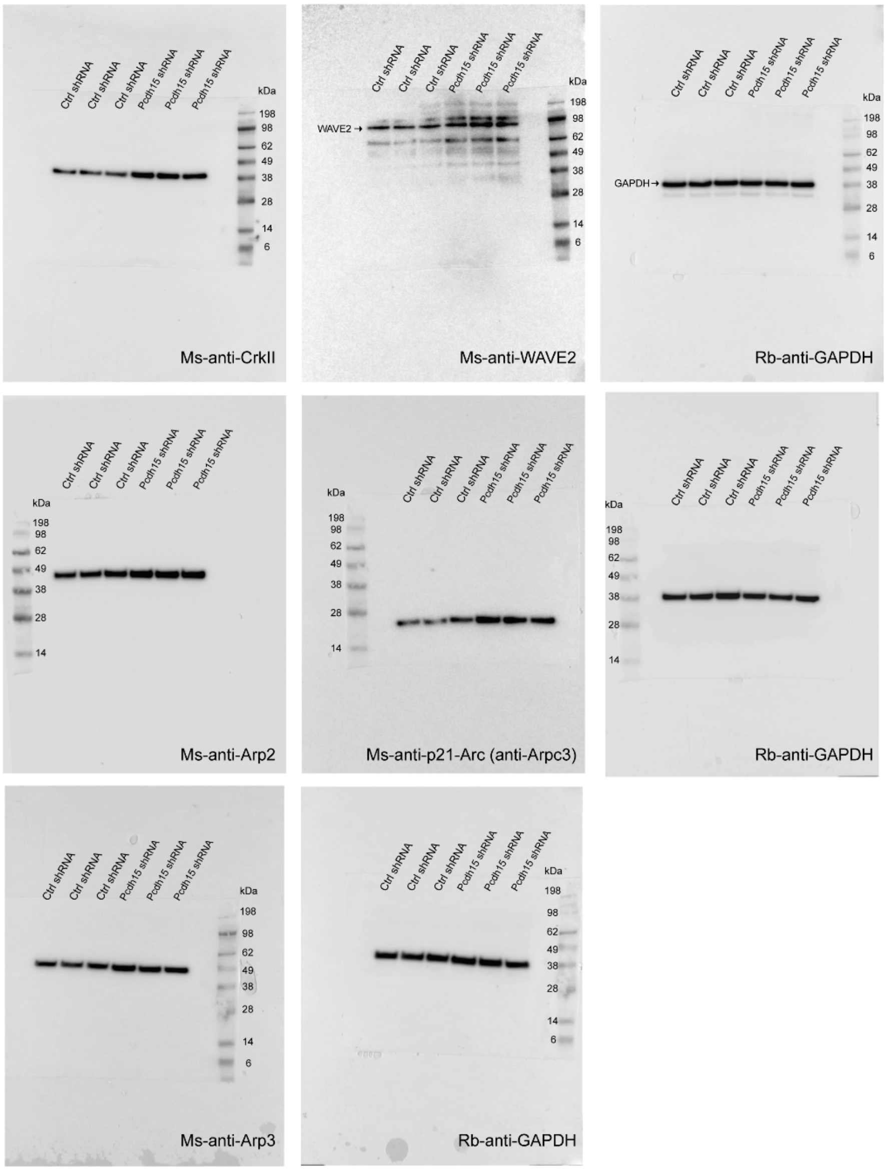

Supplementary Figure 4: Uncropped images corresponding to western blots in the main figures.

Each western blot image has been labelled to identify the figure that it relates to in the main manuscript, the protein lysates loaded, and the antibody used on the blot. The protein size markers are also labelled. When blots were stripped and re-probed, the related images are shown in the same row.
