# Supplementary material for: Protocadherin 15 suppresses oligodendrocyte progenitor cell proliferation and promotes motility through distinct signalling pathways
Source: Commun Biol. 2022 May 30;5:511. doi: 10.1038/s42003-022-03470-1 (PMC9151716; doi:10.1038/s42003-022-03470-1)
Supplement: Supplementary file 4 — Description of Additional Supplementary Files [file 42003_2022_3470_MOESM4_ESM.pdf]

Description of Additional Supplementary Files

File name: Supplementary Data 1
Description: Underlying source data for all graphs and charts.
